# The comparison of non-vitamin K antagonist oral anticoagulants versus well-managed warfarin with a lower INR target of 1.5 to 2.5 in Asians patients with non-valvular atrial fibrillation

**DOI:** 10.1371/journal.pone.0213517

**Published:** 2019-03-18

**Authors:** Yi-Hsin Chan, Kuang-Tso Lee, Yi-Wei Kao, Chien-Ying Huang, Yung-Lung Chen, Samuel Chi-Ling Hang, Pao-Hsien Chu

**Affiliations:** 1 The Cardiovascular Department, Chang Gung Memorial Hospital, Linkou, Taoyuan, Taiwan; 2 College of Medicine, Chang Gung University, Taoyuan, Taiwan; 3 Microscopy Core Laboratory, Chang Gung Memorial Hospital, Linkou, Taoyuan, Taiwan; 4 Graduate Institute of Business Administration, College of Management, Fu Jen Catholic University, Taipei, Taiwan; 5 Division of Cardiology, Department of Internal Medicine, Kaohsiung Chang Gung Memorial Hospital, Kaohsiung, Taiwan; Massachusetts General Hospital, UNITED STATES

## Abstract

**Background:**

Previous studies indicated low-intensity warfarin (INR target of 1.5–2.5) achieved reduced hemorrhage without increasing thromboembolism for Asians with non-valvular atrial fibrillation (NVAF). Whether non-vitamin K antagonist oral anticoagulant (NOAC) is superior to warfarin with good time in the therapeutic range (TTR) based on lower INR target among Asians with NVAF remains unknown.

**Methods:**

In this retrospective study collected from Taiwan Chang Gung Memorial Hospital Database, there were 5,197, 3,396, and 9,898 consecutive patients taking warfarin, NOAC, and no-treatment, respectively, from January 1, 2000 to December 31, 2015. Propensity-score weighting was used across the study groups. Patients were followed until the first occurrence of study outcome or end date of study.

**Results:**

Among those patients taking warfarin, the mean”artificial” TTR (aTTR) based on a lower INR target of 1.5–2.5 was 44.4±33.3%. Total 79.2% (n = 2,690) patients took low-dose NOACs. Patients with aTTR in the range from <30%(34.0%), 30–50%(17.6%), 50–70%(23.5%) to >70%(24.9%) showed decremental risks of efficacy and composite outcome compared with no-treatment. The risk of major bleeding didn’t increase among patients with top aTTR>70% compared to no-treatment. The NOAC group showed a comparable risk of composite outcome to the warfarin subgroup with aTTR of >70% (*P* = 0.485). The NOAC group had a lower risk of composite outcome than warfarin subgroup with TTR of>70% based on the INR target of 2.0–3.0 (*P* = 0.004).

**Conclusions:**

NOACs showed a comparable risk of efficacy, safety, and composite outcome to well-managed warfarin based on a lower INR target of 1.5–2.5 in Asians with NVAF taking oral anticoagulants.

## Introduction

Warfarin is commonly used for prevention of thromboembolic events in patients with non-valvular atrial fibrillation (NVAF). Previous meta-analysis indicated that warfarin reduced the risk of thromboembolic events by 65% and all-cause mortality by 22% as well when compared with no treatment [1]. However, the benefit of warfarin was largely compromised by its inconvenience to use and increased risk of major bleeding. The risks of bleeding and thromboembolism depend on the intensity of anticoagulation as measured by the International Normalized Ratio (INR) when taking warfarin. Both the European Society of Cardiology (ESC) and the American Heart Association (AHA) recommend a target of INR range of 2.0 to 3.0 for prevention of thromboembolism in patients with NVAF [2, 3], where the lowest risk of thromboembolism and bleeding cab be only achieved in such a narrow therapeutic range. However, several studies indicated that Asians are more sensitive to warfarin and vulnerable to warfarin related bleeding than Non-Asians [4, 5]. The meta-analysis indicated that low-intensity warfarin therapy (INR target of 1.5–2.5) can achieve reduced hemorrhage without increasing thromboembolism for Asian patients with NVAF taking warfarin [6–9]. Recently, non-vitamin K antagonist oral anticoagulants (NOACs) have been demonstrated to be effective and safe for prevention of thromboembolism in patients with NVAF [10]. It is noted that NOACs were more effective and safer in Asians than in non-Asians, which was majorly contributed from the tendency of poorer TTRs (time in therapeutic range) with the INR target of 2.0–3.0 among Asians taking warfarin [11]. However, the potential benefit of NOACs over warfarin with high TTR based on a lower INR target of 1.5–2.5 remained questionable among Asians with NVAF. This study aimed at elucidating the efficacy and safety of NOACs compared to warfarin with a variety of TTR with a lower INR target of 1.5–2.5 specifically focused on Asians with NVAF taking oral anticoagulant.

## Methods

### I. Study population

This was an observational study based on a hospital-based AF registry. This study was approved by the Institutional Review Board of Chang Gung Memorial Hospital. The Chang Gung Memorial Hospital system was the largest medical group in Taiwan, which contained 6 medical centers and provided around 11% of total mandatory universal health insurance service in Taiwan. Informed consents were not obtained from the patients due to the registry nature of the study; however, all patient information or records was de-identified and anonymized to protect patient’s privacy before analysis.

### II. Study design

**Fig 1:**
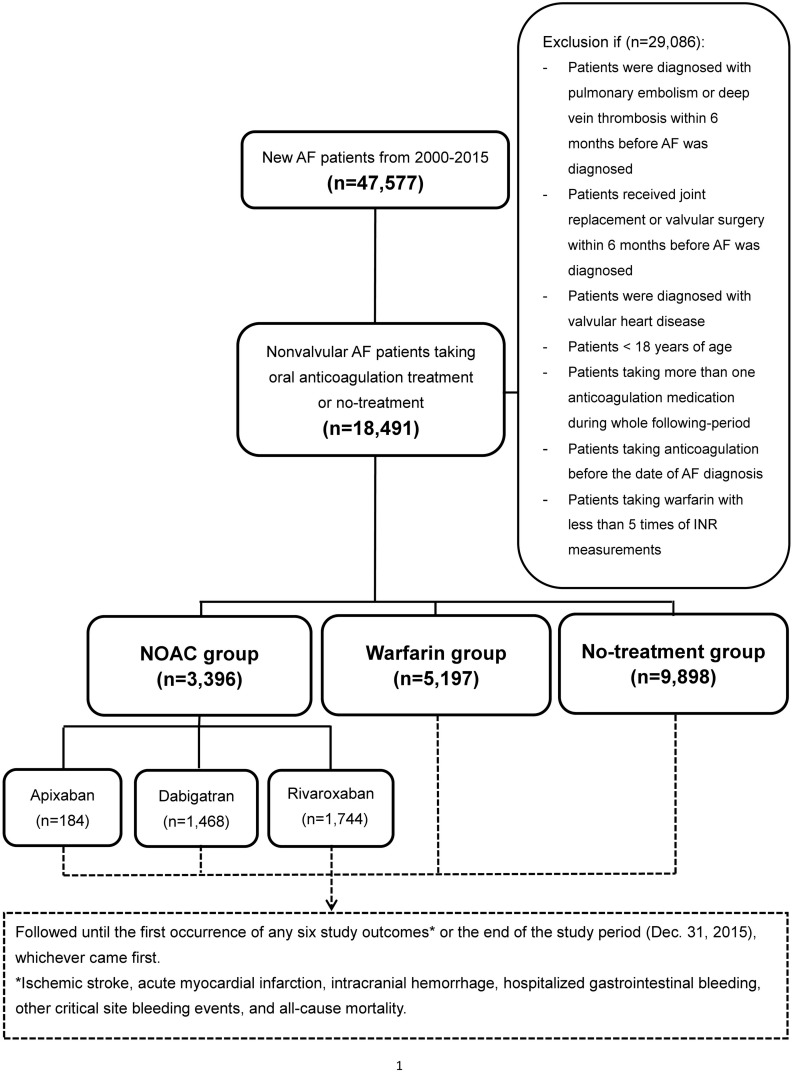
Enrollment of patients with non-valvular atrial fibrillation (NVAF). From January 1, 2000, to December 31, 2015, 5,197, 3,396, and 9,898 NVAF patients taking warfarin, non-vitamin K antagonist oral anticoagulant (NOAC), and no-treatment were enrolled in this study.

A dynamic cohort with three study groups (NOAC, warfarin, and no-treatment) was used in the study. A flowchart of the study enrollment is shown in **Fig 1**. A total of 47,577 patients diagnosed with AF (*International Classification of Diseases (the ninth revision) Clinical Modification (ICD-9-CM)* codes (427.31) from January 1, 2000 to December 31, 2015 were identified. For the NOAC group, those patients taking first prescription of a NOAC including dabigatran (approval date: June 1, 2012), rivaroxaban (approval date: February 1, 2013), or apixaban (approval date: June 1, 2014) were enrolled in the NOAC group. The index date was defined as the first date of prescription for any NOAC after June 1, 2012. Those NOAC patients with any previous warfarin exposure were excluded from the analysis. For the non-treatment group, those patients without any anticoagulation therapy during the whole following-up period were enrolled for control group. The index date was defined as the diagnosis of AF for the non-treatment group. For the warfarin group, those patients taking warfarin with at least 5 consecutive measurements of INR available until the end date of study were enrolled as the warfarin group. The index date was defined as the fifth date of INR measurements due to the large fluctuation in measurements during initial warfarin adjustment.

### III. The warfarin group, INR measurements and TTR

All INR readings after the index date of the warfarin group were included in the present analysis. Those patients with any interval of two consecutive INR reading exceeding 90 days were excluded. Implausible INR values of less than 0.8 or greater than 12 were also excluded. The TTR during the whole following period of each patient taking warfarin were obtained by using the Rosendaal method, in which INR was assumed to change in a linear manner between measurements, and INR values on the days with no measurement were interpolated [12]. TTR was estimated using INR readings until discontinuation or interruption of warfarin, an outcome event, or the end of follow-up. Therefore, INR values after any study outcome were not used to study the relationship between TTR and any study outcome. In line with current guidelines, we believe most clinicians would have been aiming for an INR target range of 2.0 to 3.0. Therefore to explore TTRs in a range of 1.5 to 2.5, we artificially created these TTRs in a post-hoc analysis and have labeled these TTR based a lower INR target as the “artificial” TTR (aTTR) in the present study.

### IV. Study outcomes

The follow-up period was defined from the index date until the first occurrence of any study outcome, discontinuation or interruption of warfarin/NOAC, or the end date of study period (December 31, 2015), whichever came first. The efficacy outcome was defined by the summation of ischemic stroke, systemic embolism, acute myocardial infarction, and mortality, while the safety outcome was defined by the summation of intracranial hemorrhage, gastrointestinal bleeding, and other critical site bleeding. All study outcomes were required to be a discharge diagnosis. The composite outcome was defined as the summation of all efficacy and safety outcomes. The ICD-9-CM codes used to identify the study outcome and the baseline covariates are summarized in **S1 Table.**

### V. Exclusion criteria

Patients were excluded who have less than 5 times of INR measurements during their entire treatment course. To establish a cohort of NVAF patients who took oral anticoagulant for the primary purpose of stroke prevention, those patients were excluded with diagnoses indicating venous thromboembolism (pulmonary embolism or deep vein thrombosis), valvular atrial fibrillation (mitral stenosis or valvular surgery), or joint replacement therapy within 6 months before the index date.

### VI. Covariates

Baseline covariates were referred to any claim record with the above diagnoses or medication codes prior to the index date. A bleeding history was confined to events within 6 months preceding the index date. A history of prescription for medicine was confined to at least once within 3 months preceding the index date. The CHA_2_DS_2_-VASc score (congestive heart failure, hypertension, age 75 years or older, diabetes mellitus, previous stroke or transient ischemic attack, vascular disease, age 65 to 74 years, female gender) was adopted to predict the risk of ischemic stroke/thromboembolic events in patients with NVAF, and the HAS-BLED score (hypertension, abnormal renal or liver function, stroke, bleeding history, labile INR, age 65 years or older, and antiplatelet drug or alcohol use) was adopted to predict the risk of bleeding in NVAF patients treated with oral anticoagulant [13, 14].

### VII. Statistical analysis

We used the propensity score method to simulate the effect of randomized clinical trial for observational cohort data and to estimate the six study outcomes of three study groups [15]. Inverse probability of treatment weights of propensity scores was used to balance covariates across the four groups. The weights were derived to obtain estimates representing average treatment effects in the treated. The covariates in **Table 1** were included in the propensity models, except for CHA_2_DS_2_-VASc and HAS-BLED scores, because CHA_2_DS_2_-VASc and HAS-BLED scores were a combination of other covariates. Incidence rates were estimated using the total number of study outcomes during the follow-up period divided by person-years at risk. The risk of time-dependent study outcomes for three study groups was obtained using survival analysis (Kaplan-Meier method and log-rank test for univariate analysis and Cox proportional hazards regression for multivariate analysis). The balance of covariates at baseline among study groups was assessed using the absolute standardized mean difference (ASMD) rather than statistical testing, because balance is a property of the sample and not of an underlying population. Another advantage of using ASMD is not influenced by sample size. The value of ASMD ≤ 0.1 indicates a negligible difference in potential confounders between the two study groups (**Tables 1** and **2**). Statistical significance was defined as a *P-*value < 0.05. All statistical analyses were performed using SAS 9.4 (SAS Institute Inc., Cary, NC, USA).

**Table 1:** Baseline characteristics of non-valvular atrial fibrillation (NVAF) patients taking NOAC, warfarin, and no-treatment before propensity score weighting.

	NOA C n = 3,396	Warfarin n = 5,197	No-treatment n = 9,898	ASMD NOAC vs. No-treatment	ASMD Warfarin vs. No-treatment
Age, yrs	74.4±10.2	66.0±12.5	71.3±12.9	0.259	0.425
< 65 (%)	15.5%	42.5%	26.5%		
65~74 (%)	29.9%	30.0%	27.3%		
75~84 (%)	39.5%	22.7%	32.4%		
> 85 (%)	15.1%	4.8%	13.9%		
Male (%)	58.7%	55.6%	58.6%	0.002	0.058
CHA2DS2-VASc	4.45±1.88	3.38±2.14	3.36±2.00	0.559	0.017
HAS-BLED	3.82±1.08	3.21±1.42	3.31±1.09	0.474	0.072
Chronic lung disease	12.9%	7.9%	13.7%	0.026	0.192
Chronic liver disease	18.1%	12.8%	14.0%	0.112	0.031
Chronic kidney disease	20.3%	14.6%	17.7%	0.066	0.084
Congestive heart failure	26.2%	18.1%	19.0%	0.173	0.023
Hypertension	65.9%	47.5%	41.9%	0.496	0.120
Hyperlipidemia	38.1%	27.2%	20.1%	0.401	0.173
Diabetes mellitus	32.4%	25.7%	23.6%	0.195	0.055
Previous stroke	28.1%	19.8%	12.4%	0.402	0.208
Previous TIA	4.1%	2.5%	1.3%	0.170	0.094
Myocardial infarction	10.8%	7.5%	12.6%	0.060	0.163
Gout	15.8%	12.5%	10.0%	0.170	0.082
Peripheral artery disease	0.2%	0.3%	0.4%	0.030	0.001
Malignancy	13.7%	8.7%	15.7%	0.056	0.209
History of bleeding	19.9%	11.9%	22.2%	0.062	0.273
Use of antiplatelet agent	20.9%	25.4%	48.2%	0.597	0.485
Use of NSAIDs	60.2%	39.1%	41.4%	0.385	0.042
Use of PPI	39.0%	19.8%	25.0%	0.298	0.125
Use of H2 blocker	52.9%	32.5%	37.5%	0.311	0.102
Use of ACEI/ARB	76.6%	55.8%	41.3%	0.767	0.298
Use of amiodarone	37.9%	28.1%	16.7%	0.487	0.283
Use of beta-blocker	73.2%	51.1%	39.1%	0.730	0.245
Use of diltiazem/verapamil	41.2%	32.3%	23.1%	0.395	0.213
Use of digoxin	33.7%	41.1%	12.1%	0.531	0.693
Use of statin	43.3%	25.1%	17.6%	0.576	0.188
PCI	7.5%	4.6%	8.2%	0.031	0.147
CABG	1.0%	1.1%	1.9%	0.085	0.066

ACEI = angiotensin-converting-enzyme inhibitor; AF = atrial fibrillation; ARB = angiotensin II receptor antagonists; CABG = coronary artery bypass graft; CHA_2_DS_2_-VASc = congestive heart failure, hypertension, age 75 years or older, diabetes mellitus, previous stroke/transient ischemic attack, vascular disease, age 65 to 74 years, female; HAS-BLED = hypertension, abnormal renal or liver function, stroke, bleeding history, labile INR, age 65 years or older, and antiplatelet drug or alcohol use. Labile INR could not be determined from claims and was excluded from our scoring; PCI = percutaneous coronary intervention; PPI = proton pump inhibitor; NOAC = non-vitamin K oral antagonist; NSAIDs = non-steroid anti-inflammatory drugs; TIA = transient ischemic attack

## Results

A total of 5,197, 3,396, and 9,898 consecutive patients taking warfarin, NOAC, and no-treatment, respectively, from January 1, 2000 to December 31, 2015, were enrolled. In general, NOAC patient group was older, had higher CHA_2_DS_2_-VASc and HAS-BLED scores, and had a higher proportion of comorbidities and multiple medications than the warfarin and no-treatment groups before propensity score weighting (**Table 1**). After propensity score weighting, the three study groups were well-balanced in most characteristics (**Table 2**).

**Table 2:** Baseline characteristics of non-valvular atrial fibrillation (NVAF) patients taking NOAC, warfarin, and no-treatment after propensity score weighting.

	NOAC n = 3,396	Warfarin n = 5,197	No-treatment n = 9,898	ASMD NOAC vs. No-treatment	ASMD Warfarin vs. No-treatment
Age, yrs	71.6±11.4	70.1±11.7	70.3±14.1	0.100	0.015
< 65 (%)	22.7%	29.4%	28.60%		
65~74 (%)	32.8%	30.7%	26.20%		
75~84 (%)	32.5%	31.1%	32.00%		
> 85 (%)	11.0%	8.8%	13.10%		
Male (%)	57.9%	58.9%	57.60%	0.005	0.025
CHA2DS2-VASc	3.73±1.90	3.74±2.10	3.67±2.08	0.032	0.034
HAS-BLED	3.50±1.15	3.56±1.34	3.43±1.12	0.058	0.098
Chronic lung disease	11.8%	11.1%	11.4%	0.012	0.011
Chronic liver disease	16.6%	16.0%	15.1%	0.044	0.025
Chronic kidney disease	18.7%	19.8%	18.1%	0.017	0.043
Congestive heart failure	21.2%	21.1%	20.1%	0.027	0.026
Hypertension	49.4%	52.5%	49.8%	0.008	0.053
Hyperlipidemia	26.5%	29.5%	26.9%	0.009	0.059
Diabetes mellitus	26.8%	29.5%	27.3%	0.011	0.049
Previous stroke	19.6%	18.4%	18.8%	0.020	0.010
Previous TIA	2.6%	2.2%	2.3%	0.019	0.004
Myocardial infarction	9.5%	12.9%	11.0%	0.051	0.058
Gout	12.2%	13.0%	12.0%	0.007	0.030
Peripheral artery disease	0.1%	0.4%	0.3%	0.030	0.013
Malignancy	13.8%	13.3%	13.1%	0.022	0.007
History of bleeding	19.6%	19.8%	18.6%	0.024	0.029
Use of antiplatelet agent	39.1%	37.1%	35.6%	0.074	0.031
Use of NSAIDs	49.7%	46.9%	44.6%	0.103	0.045
Use of PPI	29.8%	28.1%	27.8%	0.044	0.006
Use of H2 blocker	43.2%	41.5%	40.6%	0.052	0.017
Use of ACEI/ARB	54.7%	56.3%	55.0%	0.007	0.027
Use of amiodarone	25.5%	25.9%	26.1%	0.013	0.004
Use of beta-blocker	52.5%	53.2%	51.4%	0.021	0.036
Use of diltiazem/verapamil	31.6%	30.9%	31.4%	0.005	0.011
Use of digoxin	22.7%	24.6%	26.6%	0.089	0.045
Use of statin	27.0%	28.0%	26.2%	0.016	0.039
PCI	6.3%	9.3%	7.5%	0.048	0.066
CABG	0.8%	2.1%	1.5%	0.075	0.042

The abbreviations as in **Table 1**.

### I. The clinical events, efficacy and safety outcomes of three patient groups

**Fig 2:**
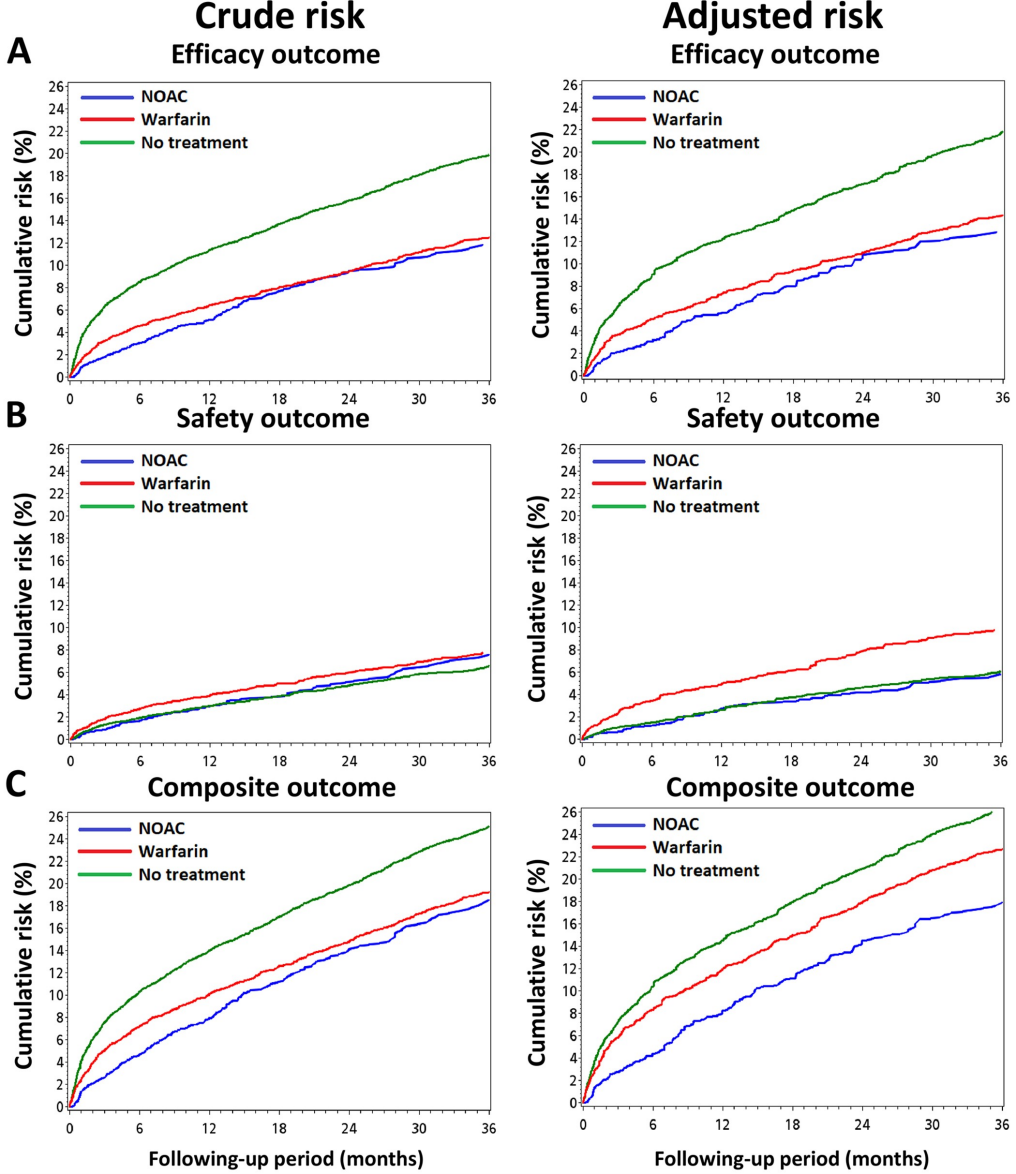
Cumulative incidence curves of outcomes for patients according to initiated treatment before and after propensity score weighting method. The NOAC and warfarin groups both showed a lower cumulative risk of efficacy outcomes compared with the no-treatment group **(A)**. Warfarin group has the highest cumulative risk of safety outcome, while the no-treatment had the lowest cumulative risk of safety event **(B)**. The weighted cumulative risk of composite outcome showed a clear separation of event curves within the three groups, in which the NOAC group had the lowest risk of composite outcome, while the no-treatment group had the highest risk of composite outcome **(C)**.

**Fig 2** showed the efficacy, safety, and composite outcome among three study groups before and after propensity score weighing adjustment. The median following up period for NOAC, warfarin, and non-treatment groups were 388, 840, and 639 days, respectively. The median adherence rate of NOACs was 83% (range 19%–100%) during the whole following period. The weighted event curves showed that NOAC and warfarin groups both had a lower cumulative risk of efficacy events compared with no-treatment group. Both warfarin (5.88%/year) and NOAC (5.51%/year) groups has a significantly lower annual incidence of efficacy outcome compared to no-treatment (9.40%/year) (Both *P* < 0.001 vs. no-treatment). For the weighted event curves of safety outcome, the warfarin group had the highest risk of safety outcome (3.92%/year), followed by those of the NOAC (2.26%/year) and no-treatment groups (2.22%/year) (*P* < 0.001 for warfarin vs. NOAC; *P* = 0.904 for NOAC vs. no-treatment). The weighted cumulative risk of composite outcome showed a clear separation of event curves within the three groups, in which the NOAC group had the lowest risk of composite outcome, while the no-treatment group had the highest risk of composite outcome **(Fig 2)**. The annual incidence of composite outcome for NOAC, warfarin, and non-treatment group were 7.76%/year, 9.79%/year, and 11.62%/year, respectively (*P* < 0.001 for NOAC vs. warfarin; *P* < 0.001 for warfarin vs. no-treatment).

### II. The efficacy and safety outcomes of warfarin group categorized by different “artificial” TTR (aTTR) based on the proportion of time spent within INR range of 1.5–2.5

**Fig 3:**
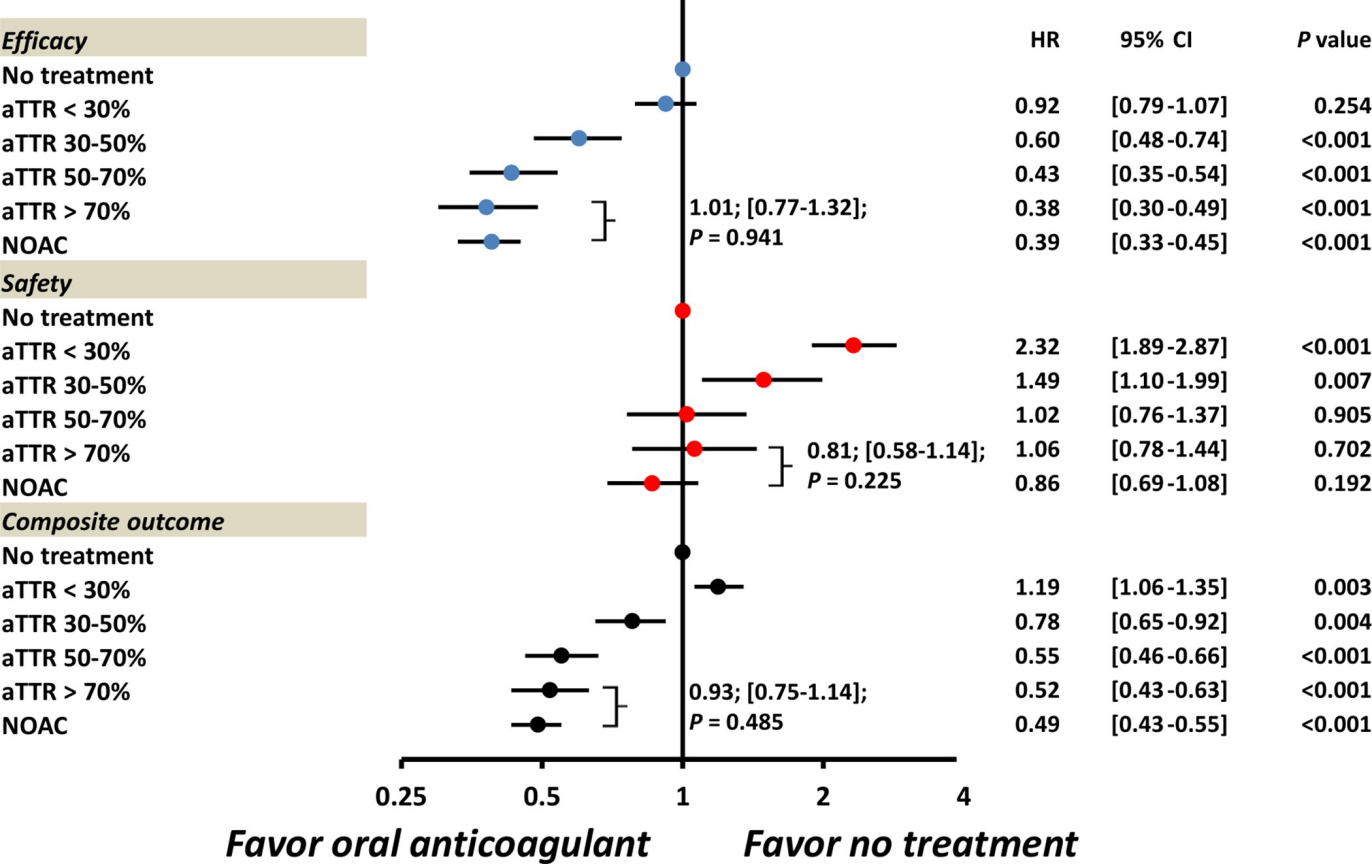
The adjusted risk of outcomes for patients taking oral anticoagulants in relative to no-treatment according to NOAC and warfarin with different “artificial” TTR (aTTR) based on a lower INR target of 1.5–2.5. In general, the warfarin group with aTTR in the range from < 30%, 30–50%, 50–70% to > 70% showed a decremental risk of efficacy, safety, and composite outcomes compared with the no-treatment group. The NOAC group showed comparable risks of efficacy, safety, and composite outcome to the warfarin subgroup with aTTR of > 70%. The adjusted factors were the all covariates listed in the **Table 1**.

We calculate the aTTR, which reflected the proportion of time spent within therapeutic range of 1.5 to 2.5, in those 5,197 patients taking warfarin. The mean aTTR for the warfarin group was 44.4 ± 33.3%. There were 34.0% (n = 1,768), 17.6% (n = 913), 23.5% (n = 1,220), and 24.9% (n = 1,296) patients with the aTTR range of < 30%, 30–50%, 50–70%, and > 70% during the following-up period, respectively. **Table 3** and **Fig 3** summarizes the relative risk of efficacy, safety, and composite endpoints for those patients taking oral anticoagulants compared to no-treatment according to NOACs and warfarin with different aTTR. The warfarin group with aTTR in the range from < 30%, 30–50%, 50–70%, to > 70% showed a decremental risk of efficacy outcomes compared with no-treatment. The NOAC group showed a comparable efficacy outcome to the warfarin group with aTTR of > 70% (adjusted hazard ratio (HR): 1.01, [95% confidential interval (CI): 0.77–1.32]; *P* = 0.941 for NOAC vs. aTTR of > 70%). For the safety outcome, those warfarin groups with aTTRs in the range of < 30% (adjusted HR: 2.32, [95% CI: 1.89–2.87]) and 30–50% (adjusted HR: 1.49, [95% CI: 1.10–1.99]) were both associated with a higher risk of major bleeding, while aTTR in the range of 50–70%, >70%, as well as the NOAC group didn’t cause a higher risk of major bleeding compared with no-therapy group (adjusted HR: 0.86, [95% CI: 0.69–1.08] for NOAC group). For the composite outcomes, aTTR in the range of > 70% were associated with the lowest incidence of composite events (adjusted HR: 0.52, [95% CI: 0.75–1.14]; *P* < 0.001 for TTR of > 70%). Importantly, the NOAC group showed a comparable risk of composite outcome to the warfarin group with top aTTR of > 70% (adjusted HR: 0.93, [95% CI: 0.75–1.14]; *P* = 0.485 for NOAC vs. aTTR of > 70%). We compared the risks of ischemic stroke/systemic embolism and critical care bleeding for NOACs vs. warfarin. The results showed that the NOAC group showed comparable risks of ischemic stroke/systemic embolism and critical care bleeding to the warfarin group with aTTR of > 70% (**S1 Fig**). Among the NOAC group, there were 5.4% (n = 184), 43.2% (n = 1,468), and 51.4% (n = 1,744) patients taking apixaban, dabigatran, and rivaroxaban, respectively. Total of 79.2% patients (n = 2,690) took the low-dose NOACs, which was defined as the use of apixaban 2.5 mg twice daily, dabigatran 110 mg twice daily, or rivaroxaban 15–10 mg once daily. The result showed that both low-dose and standard-dose NOAC was associated with comparable risks of efficacy, safety, and composite outcome to the warfarin group with aTTR of > 70% after adjustment (**S2 Fig)**. We also compared three different NOACs versus warfarin with a variety of aTTR. The result showed that apixaban, dabigatran, and rivaroxaban all have comparable risks of efficacy, safety, and composite outcome to the warfarin group with aTTR of > 70% after adjustment (**S3 Fig)**.

**Table 3:** The adjusted risk of outcomes for those patients taking oral anticoagulants in relative to no-treatment according to NOAC and warfarin with different TTR.

	Efficacy		Safety		Composite outcome	
	Adjusted* HR (95% CI)	*P* value	Adjusted* HR (95% CI)	*P* value	Adjusted* HR (95% CI)	*P* value
**“Artificial” TTR (aTTR) base on the INR target of 1.5–2.5**						
**aTTR < 30%**	0.92 (0.79–1.07)	0.254	2.32 (1.89–2.87)	< 0.001	1.19 (1.06–1.35)	0.003
**aTTR 30–50%**	0.60 (0.48–0.74)	< 0.001	1.49 (1.10–1.99)	0.007	0.78 (0.65–0.92)	0.004
**aTTR 50–70%**	0.43 (0.35–1.54)	< 0.001	1.02 (0.76–1.37)	0.905	0.55 (0.46–0.66)	< 0.001
**aTTR > 70%**	0.38 (0.30–0.49)	< 0.001	1.06 (0.78–1.44)	0.702	0.52 (0.43–0639)	< 0.001
**NOAC**	0.39 (0.33–0.45)	< 0.001	0.86 (0.69–1.08)	0.192	0.49 (0.43–0.55)	< 0.001
**TTR base on the INR target of 2.0–3.0**						
**TTR < 30%**	0.61 (0.53–0.69)	< 0.001	1.70 (1.40–2.05)	< 0.001	0.82 (0.74–0.92)	< 0.001
**TTR 30–50%**	0.67 (0.55–0.82)	< 0.001	1.14 (0.84–1.55)	0.411	0.77 (0.65–0.92)	0.003
**TTR 50–70%**	0.50 (0.38–0.66)	< 0.001	1.31 (0.91–1.88)	0.152	0.66 (0.53–0.82)	< 0.001
**TTR > 70%**	0.52 (0.38–0.72)	< 0.001	1.47 (0.98–2.20)	0.064	0.71 (0.55–0.91)	0.007
**NOAC**	0.38 (0.33–0.45)	< 0.001	0.85 (0.68–1.06)	0.149	0.48 (0.42–0.55)	< 0.001

CI = Confidential interval; HR = Hazard ratio; INR = International Normalized Ratio; NOAC = non-vitamin K oral antagonist; TTR = time in therapeutic range

*The adjusted factors were the all covariates listed in the **Table 1**.

Sensitivity analysis was performed by restricting the patient enrollment from January 1, 2010 to December, 31, 2015. A total of 3,195, 3,396, and 4,878 consecutive patients taking warfarin, NOAC, and no-treatment, respectively, from January 1, 2010 to December 31, 2015, were enrolled. There were 36.6% (n = 1,170), 16.2% (n = 519), 20.7% (n = 662), and 26.4% (n = 844) patients with the aTTR range of < 30%, 30–50%, 50–70%, and > 70% during the following-up period, respectively. The results of sensitivity analysis were compatible to those of the main analysis, in which the NOAC group showed a comparable efficacy (adjusted HR: 0.96, [95% CI: 0.70–1.32]; *P* = 0.816), safety (adjusted HR: 0.78, [95% CI: 0.53–1.16]; *P* = 0.225), and composite outcome (adjusted HR: 0.86, [95% CI: 0.68–1.10]; *P* = 0.863) to the warfarin group with aTTR of > 70% after adjustment (**S4 Fig)**.

### III. The efficacy and safety outcomes of warfarin group categorized by different TTR based on the proportion of time spent within INR range of 2.0–3.0

**Fig 4:**
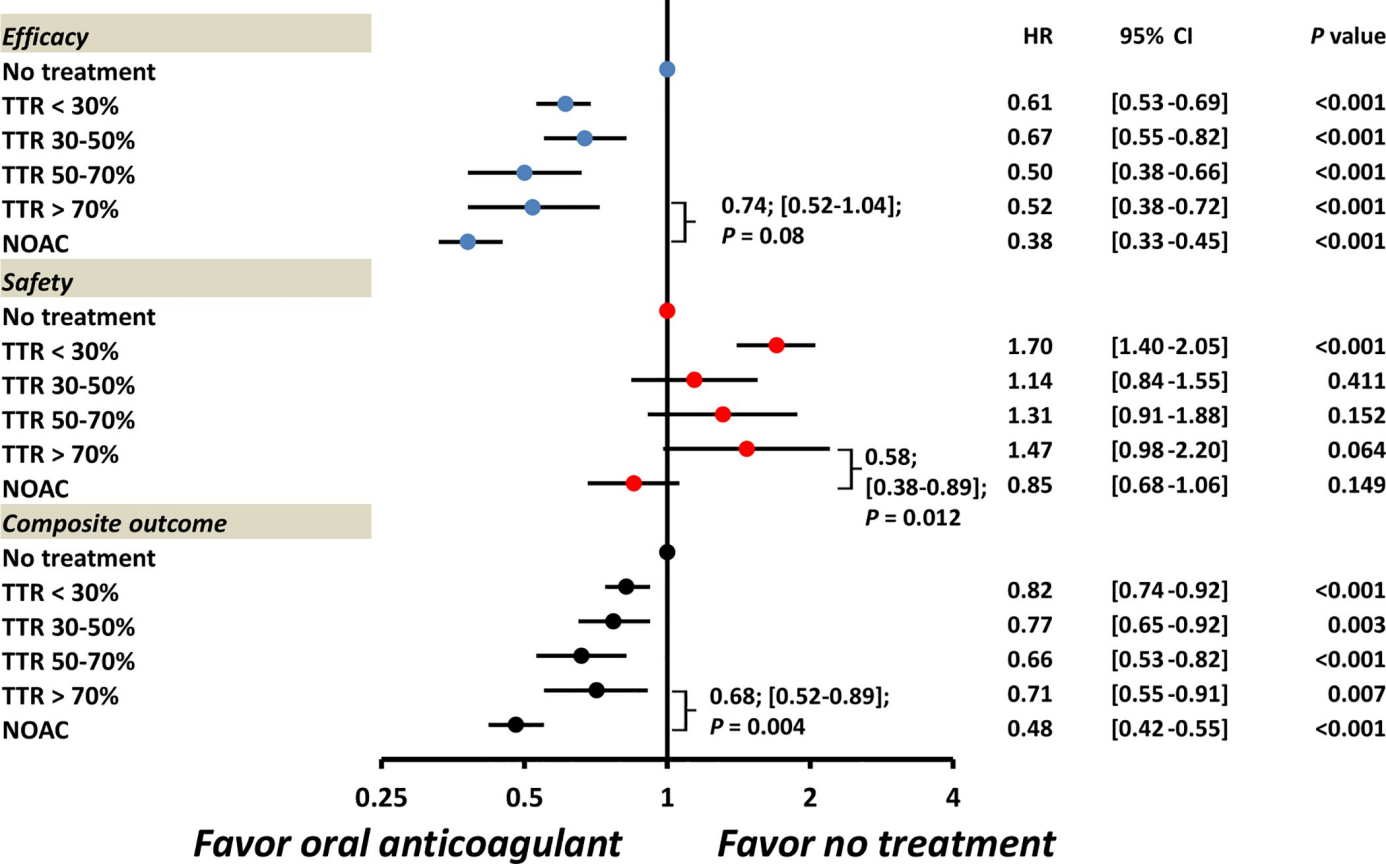
The adjusted risk of outcomes for patients taking oral anticoagulants in relative to no-treatment according to NOAC and warfarin with different TTR based on the INR target of 2.0–3.0. The NOAC group showed lower risks of safety and composite outcome to the warfarin group with TTR of > 70% based on the INR target of 2.0–3.0. The adjusted factors were the all covariates listed in the **Table 1**.

We also calculate the TTR within therapeutic range of 2.0 to 3.0 in those patients taking warfarin (**Table 3)**. The mean TTR (INR 2.0–3.0) for the warfarin group was 24.9 ± 27.7%. There were 58.5% (n = 3,038), 20.5% (n = 1067), 12.4% (n = 643), and 8.6% (n = 449) patients with the TTR range of < 30%, 30–50%, 50–70%, and > 70% during the following-up period, respectively. **Fig 4** indicated that the NOAC group was independently associated with a lower risk of composite outcome compared with warfarin group with top TTR of > 70% based on INR target of 2.0–3.0 (adjusted HR: 0.68, [95%CI: 0.52–0.89]; *P* = 0.004).

## Discussion

In the present large in-hospital cohort study, we investigated the aTTR-specific risk of thromboembolic and major bleeding events in Asians with NVAF taking moderate-intensity anticoagulation therapy with the INR target of 1.5–2.5. The adjusted risk of efficacy, safety, and composite outcomes in patients taking NOACs were comparable to those taking warfarin with aTTR of > 70%. Our study indicated that NOACs may be an alternative choice to warfarin with aTTR of > 70% based on a lower INR target of 1.5–2.5 in Asian patients with NVAF.

Currently, major guidelines including the AHA/ACC and ESC used in Western countries all recommended an INR target in the range of 2.0 to 3.0 for thromboembolic prevention in patients with NVAF [2, 3]. The overall guideline in Taiwan and most Asia-Pacific regions also follows that of Western societies, with the optimal INR target set in the range of 2.0 to 3.0 for thromboembolic prevention in patients with NVAF [16]. However, several studies have indicated that lower INR level may be more suitable in Chinese patients. Cheung et al. studied 555 Chinese patients taking warfarin for AF stroke prevention, and the result indicated that INR in the range of 1.5 to 3.0 was efficacious and safe for thromboembolic prevention [8]. You et al. retrospectively studied 491 Chinese patients taking warfarin with the original aim of achieving a target INR of 2.0 to 3.0 [6]. However, they observed that an INR in the range of 1.8 and 2.4 were associated with the lowest incidence of thromboembolic and bleeding events. In addition, few prospective studies with specific enrollment of Japanese patients also indicated that INR value in the range of 1.6 to 2.6 was associated with the lowest risk of thromboembolic and major bleeding events in Japanese, which make the Japanese guideline suggested a lower INR range of 1.6 to 2.6 in patients over 70 years of age [17]. Furthermore, recent meta-analysis has demonstrated that low-intensity warfarin therapy (INR in the range of 1.5 to 2.5) caused reduced hemorrhage without increasing thromboembolism among East Asian patients with NVAF taking warfarin [9]. Asian patients are more prone to warfarin related major bleeding compared to non-Asians, which can be partially explained by the variations of genetic polymorphisms for VKA metabolism, multiple drug-food interaction, and use of herbal medicine [18, 19]. Those differences may explain the discrepancy of safety profile between Asians and non-Asians taking warfarin.

Previous studies indicated that patients on warfarin at low range of TTR with the INR target of 2.0–3.0 have higher risk of thromboembolic and bleeding events compared with than those with high TTR [20]. Our studies was the largest retrospective cohort with enrollment of 5,197 Asians/Chinese taking warfarin with detailed INR measurements during following-up period, also indicated that aTTR of > 70% with a lower INR target of 1.5–2.5 was associated with a lowest risk of composite outcome compared to the no-therapy group. Furthermore, those patients with aTTR of < 30% even have a worse outcome than those without any antithrombotic therapy **(Fig 3)**. Our data demonstrated the benefits of warfarin therapy for thromboembolic prevention in Asian patients with NVAF are closely dependent on the quality of anticoagulation control, as reflected by the aTTR with a lower INR target of 1.5–2.5. Further prospective randomized studies are necessary to confirm the efficacy and safety of a low-intensity warfarin in Asians with NVAF.

The introduction of NOACs has brought the innovation of stroke prevention in AF patients taking oral anticoagulants [10]. However, the benefit of NOAC over warfarin in reduction of thromboembolism and major bleeding depends on the quality of warfarin treatment; and the superiority in efficacy and safety of NOAC compared with warfarin for stroke prevention is lost above a TTR threshold of 70% based a INR target of 2.0–3.0 [21]. The post-hoc meta-analysis of those four pivotal studies of NOACs had demonstrated that NOACs were more effective and safer in Asians than in non-Asians, which was believed due to the much lower TTRs of warfarin control with the INR of 2.0–3.0 when compared with the non-Asian populations [11, 22]. However, there is no large data to compare the benefit of NOAC over warfarin with a variety of TTR control specifically focused on Asians either with the INR target of 2.0–3.0 or 1.5–2.5. Previous studies in Hong-Kong has demonstrated that dabigatran has a lower risk of ischemic stroke and intracranial hemorrhage compared with warfarin with a TTR of ≥ 65% with INR target of 2.0–3.0 in elderly Chinese with NVAF [19, 23, 24]. However, the enrollments of patient number taking warfarin or NOACs were all very limited in those studies. In our study, we demonstrated that the NOAC group, with a majority of low-dose NOACs (79.2%), has a comparable efficacy, safety, and composite outcomes to the warfarin group with aTTR of > 70% based on the INR target of 1.5–2.5 after adjustment. Further prospective and randomized studies comparing the effectiveness and safety between NOACs and well-managed warfarin in Asians need be reappraised in the future.

## Limitations

The present study had several limitations. This study is multiple-center, retrospective, and observational study, which limited the level of evidence presented. The selection of anticoagulants was not in a randomized determination, which is evidenced by significant difference in baseline characteristics between patients on no treatment, warfarin, and NOACs. Although an extensive number of variables was included in our propensity score model and achieved a close balance for most factors, residual confounding by unmeasured factors including blood pressure control, use of herbal and over the counter medications, and vitamin K dietary intake cannot be excluded due to its retrospective design. The amount of patients without any treatment is quite staggering in the study (n = 9,898). This is the largest group by quite some margin. Furthermore, the mean CHA2DS2-VASc score of the no-treatment group was 3.36 in the present study (**Table 1**), indicating that the majority of the patients in the no-treatment group was still indicated for oral anticoagulation therapy according to the current guideline. Our data was compatible to previous large national cohort study, which also reported that there were 157,829 patients (85%) indicated for oral anticoagulant (CHA_2_DS_2_-VASc score of ≥ 2) among the total 186,570 AF patients without any oral anticoagulant exposure in Taiwan’s real-world practice [25].Why such a high prevalence of patients indicated for therapy but without receiving oral anticoagulant among the total AF population especially in the Asia is unclear. One important issue is that the risk of major bleeding regarding the use of oral anticoagulant in AF patients is higher in those of Asian ethnicity [26]. Therefore, physicians in Asia may avoid prescribing any oral anticoagulants in those patients perceived to be at an increased risk of hemorrhage. Although the sensitivity analysis with restriction of the patient enrollment started from January 1, 2010 to December 31, 2015 has reduced the patient number without any treatment down to 4,878, and the result of sensitivity analysis was still compatible to those of the main analysis, this augments regarding selection bias still cannot be excluded. In addition, we observed a high prevalence of low-dose NOAC prescriptions in the present cohort. The lack of body weights and detailed renal function makes it difficult to determine if those patients taking low-dose NOACs were correctly prescribed an “adjusted” low-dose or “off-label” under-dose NOACs. Nevertheless, the present study may be highly prone to selection bias and confounded by indication as physicians may have avoided prescribing full-dose NOAC or warfarin with a target INR 2.0–3.0 in those patients perceived to be at an increased risk of bleeding. In the present study, only primary discharge diagnoses were adopted in order to improve the accuracy of clinical outcome, and some outcomes including a mild form of stroke or bleeding without hospitalization may be missed.

## Conclusions

The efficacy, safety, and composite outcome of NOACs were comparable to warfarin with aTTR of more than 70% based on a lower INR target of 1.5–2.5 in Asians/Chinese with NVAF taking oral anticoagulants. NOAC may be an effective, safe, and convenient alternative to the well-managed warfarin among those patient groups. The results are hypothesis generating and may form a basis of a randomized control trial in the future.

## Supporting information

S1 Table International classification of disease (9^th^ edition) clinical modification (ICD 9-CM) codes used to define the co-morbidities and clinical outcome in the study cohort. (PDF)

S1 Fig The adjusted risk of ischemic stroke/systemic embolism and critical care bleeding for patients taking oral anticoagulants in relative to no-treatment according to NOAC and warfarin with different aTTR based on the INR target of 1.5–2.5. The result showed that NOAC group was associated with comparable risks of ischemic stroke/systemic embolism and critical care bleeding to the warfarin group with aTTR of > 70%. The adjusted factors were the all covariates listed in the **Table 1**.
(TIFF)

S2 Fig The adjusted risk of outcomes for patients taking oral anticoagulants in relative to no-treatment according to low-dose or standard-dose NOACs and warfarin with different aTTR. The result showed that standard-dose and low-dose NOACs were both associated with comparable risks of efficacy, safety, and composite outcome to the warfarin group with aTTR of > 70%. The adjusted factors were the all covariates listed in the **Table 1**.
(TIFF)

S3 Fig The adjusted risk of outcomes for patients taking oral anticoagulants in relative to no-treatment according to three different NOACs and warfarin with different aTTR. The result showed that apixaban, dabigatran, and rivaroxaban were all associated with comparable risks of efficacy, safety, and composite outcome to the warfarin group with aTTR of > 70%. The adjusted factors were the all covariates listed in the **Table 1**.
(TIFF)

S4 Fig The adjusted risk of outcomes for patients taking oral anticoagulants in relative to no-treatment according to NOAC and warfarin with different aTTR with the enrollment from January 1, 2010 to December 31, 2015. The results of sensitivity analysis were compatible to those of the main analysis, in which the NOAC group showed a comparable efficacy, safety, and composite outcome to the warfarin group with aTTR of > 70%. The adjusted factors were the all covariates listed in the **Table 1**.
(TIF)

S1 Dataset Minimal underlying study data. (DOCX)
