# Supplementary material for: The comparison of non-vitamin K antagonist oral anticoagulants versus well-managed warfarin with a lower INR target of 1.5 to 2.5 in Asians patients with non-valvular atrial fibrillation
Source: PLoS One. 2019 Mar 18;14(3):e0213517. doi: 10.1371/journal.pone.0213517 (PMC6422299; doi:10.1371/journal.pone.0213517)
Supplement: S1 Table — (PDF) [file pone.0213517.s001.pdf]

Supplemental Materials

Supplemental Table I

International Classification of Disease (9th edition) Clinical Modification (ICD 9-CM) codes used to define the co-morbidities and clinical outcome in the study cohort

Disease	ICD-9 Codes	Diagnosis definition
Atrial fibrillation	427.31	Discharge or outpatient department ≥2
Ischemic stroke	433, 434, 436	Discharge
Systemic embolism	444	Discharge
Intracranial hemorrhage	430, 431, 432, 852, 853	Discharge
Transient ischemic attack	435	Discharge
Peripheral arterial occlusive disease	440.2	Discharge
Myocardial infarction	410, 411, 412	Discharge
Congestive heart failure	428	Discharge

Hypertension	401, 402	Outpatient department ≥2
Diabetes mellitus	250	Outpatient department ≥2
Hyperlipidemia	272	Outpatient department ≥2
Gastrointestinal bleeding	456.0, 456.2, 455.2, 455.5, 455.8, 530.7, 530.82, 531.0, 531.2, 531.4, 531.6, 532.0, 532.2, 532.4, 532.6, 533.0, 533.2, 533.4, 533.6, 534.0, 534.2, 534.4, 534.6, 569.3, 535.01, 535.11, 535.21, 535.31, 535,41, 535.51, 535.61, 535.71, 537.83, 537.84, 562.02, 562.03, 562.12 562.13 568.81, 569.3, 569.85, 578	Discharge
Other critical site bleeding	423,0, 459.0, 568.81, 593.81, 599.7, 623.8, 626.32, 626.6, 719.1, 784.7, 784.8, 786.3	Discharge
Chronic kidney disease	580-589	Outpatient department ≥2
Chronic liver disease	570, 571, 572	Outpatient department ≥2
