# Supplementary figures and images for: The comparison of non-vitamin K antagonist oral anticoagulants versus well-managed warfarin with a lower INR target of 1.5 to 2.5 in Asians patients with non-valvular atrial fibrillation
Source: PLoS One. 2019 Mar 18;14(3):e0213517. doi: 10.1371/journal.pone.0213517 (PMC6422299; doi:10.1371/journal.pone.0213517)

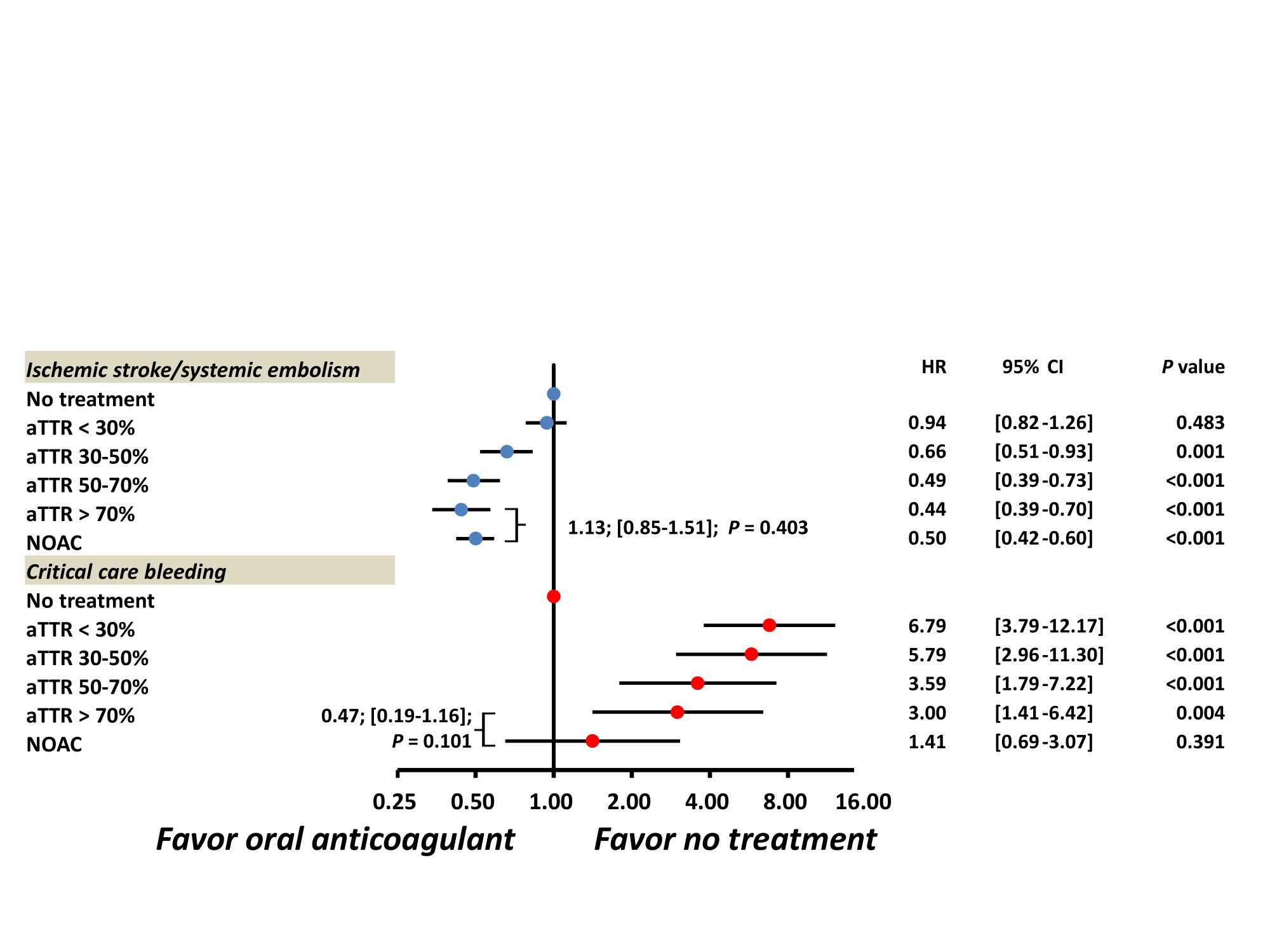

Supplement: S1 Fig — The result showed that NOAC group was associated with comparable risks of ischemic stroke/systemic embolism and critical care bleeding to the warfarin group with aTTR of > 70%. The adjusted factors were the all covariates listed in the Table 1. (TIFF) [file pone.0213517.s002.tiff]

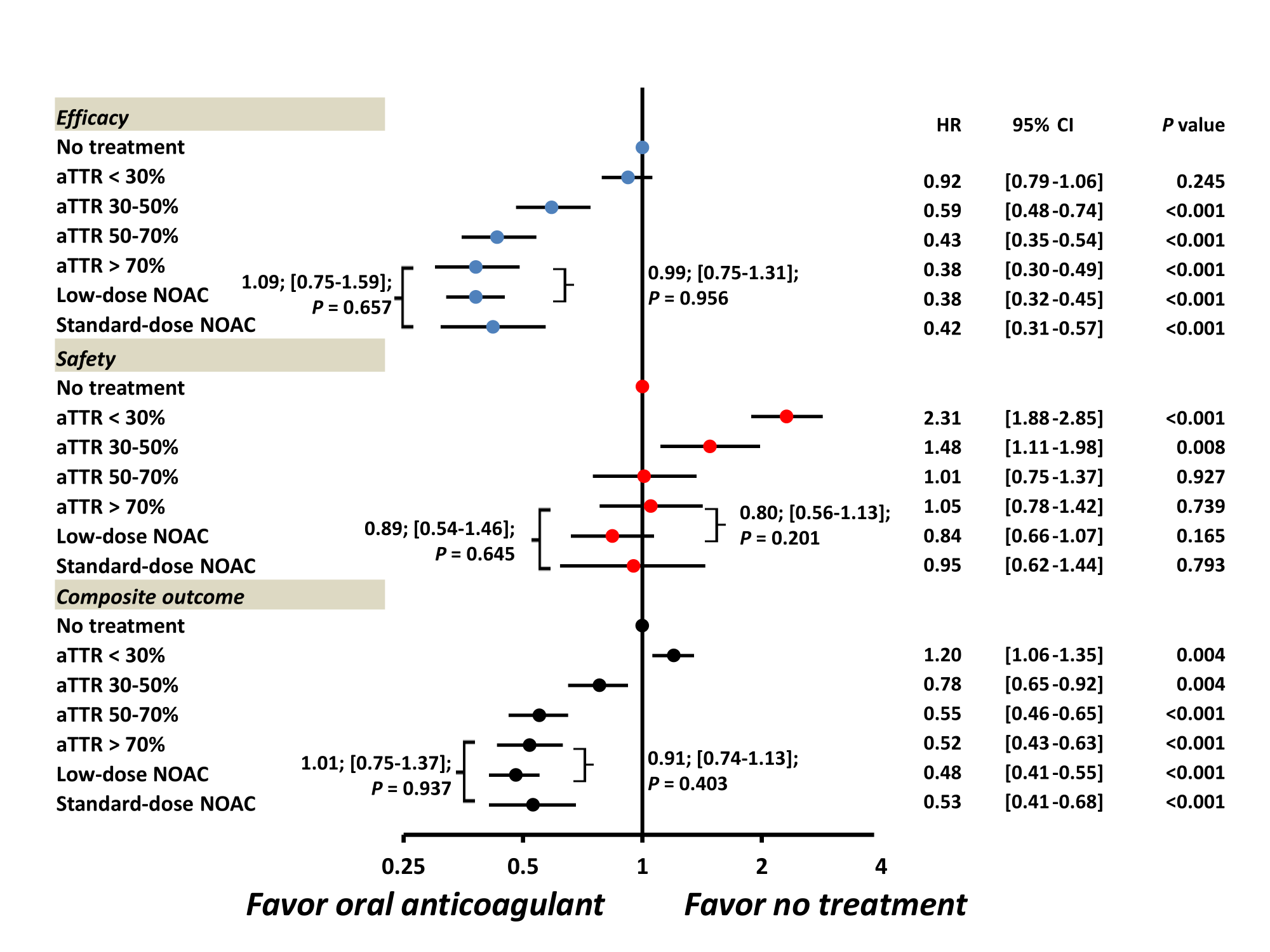

Supplement: S2 Fig — The result showed that standard-dose and low-dose NOACs were both associated with comparable risks of efficacy, safety, and composite outcome to the warfarin group with aTTR of > 70%. The adjusted factors were the all covariates listed in the Table 1. (TIFF) [file pone.0213517.s003.tiff]

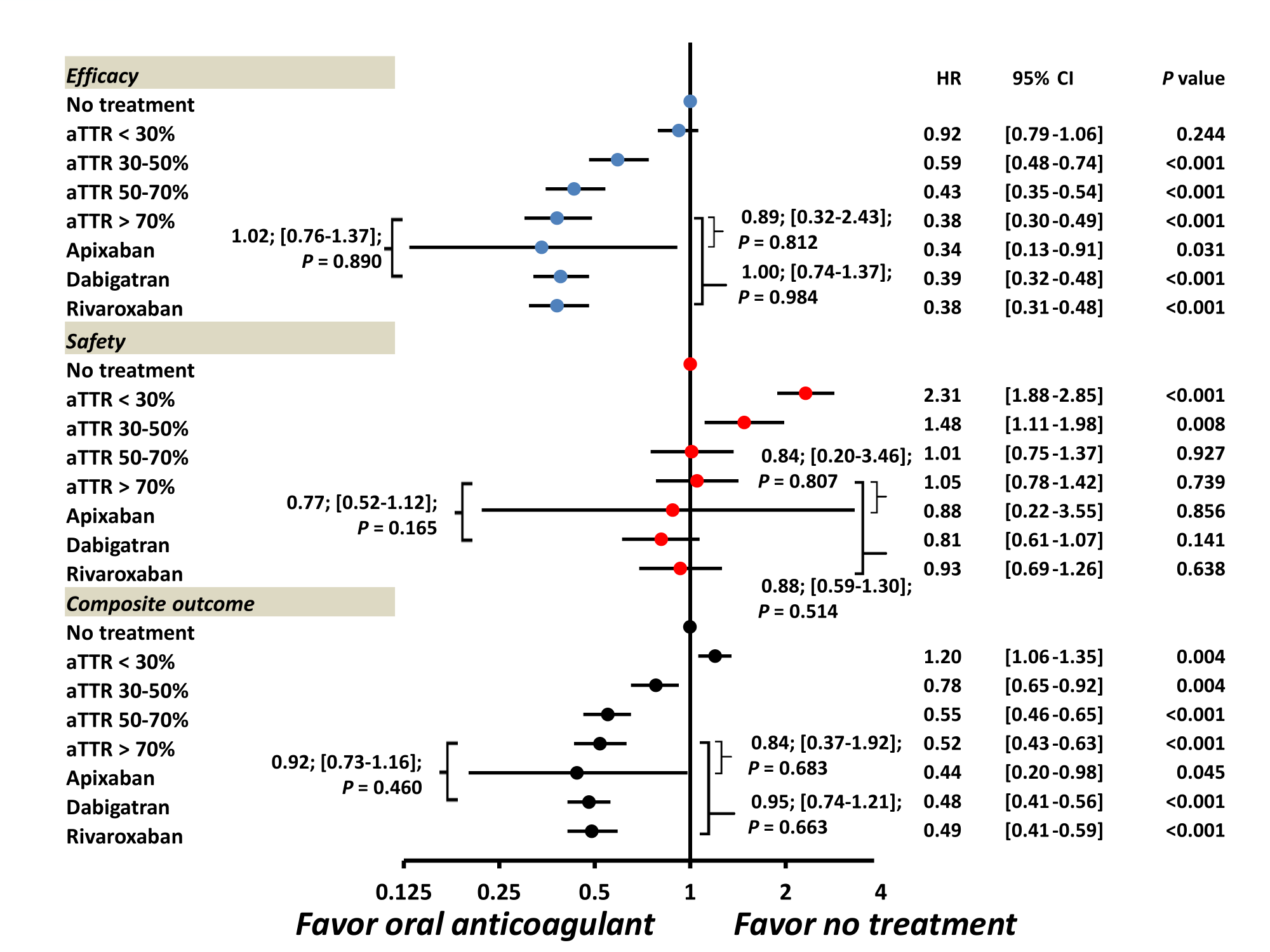

Supplement: S3 Fig — The result showed that apixaban, dabigatran, and rivaroxaban were all associated with comparable risks of efficacy, safety, and composite outcome to the warfarin group with aTTR of > 70%. The adjusted factors were the all covariates listed in the Table 1. (TIFF) [file pone.0213517.s004.tiff]

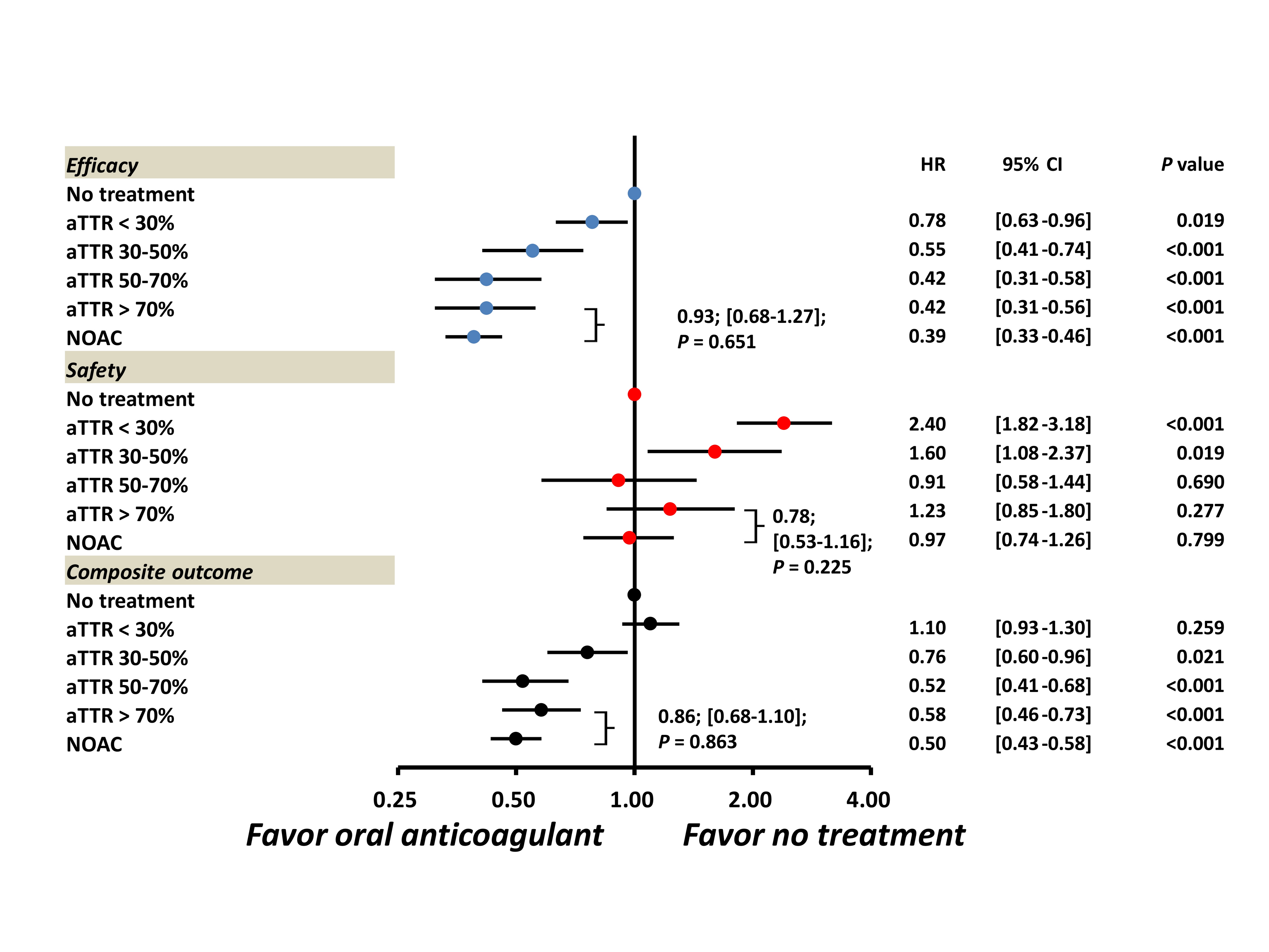

Supplement: S4 Fig — The results of sensitivity analysis were compatible to those of the main analysis, in which the NOAC group showed a comparable efficacy, safety, and composite outcome to the warfarin group with aTTR of > 70%. The adjusted factors were the all covariates listed in the Table 1. (TIF) [file pone.0213517.s005.tif]
