# Supplementary material for: The comparison of non-vitamin K antagonist oral anticoagulants versus well-managed warfarin with a lower INR target of 1.5 to 2.5 in Asians patients with non-valvular atrial fibrillation
Source: PLoS One. 2019 Mar 18;14(3):e0213517. doi: 10.1371/journal.pone.0213517 (PMC6422299; doi:10.1371/journal.pone.0213517)
Supplement: S1 Dataset — (DOCX) [file pone.0213517.s006.docx]

| **Figure 2** | **Incidence rate**  **(%/year) (Crude)** | **Incidence rate**  **(%/year) (Adjusted)** | **Statistical method** | **P value (Crude)** | **P value (Adjusted)** | **# sample** |
| --- | --- | --- | --- | --- | --- | --- |
| **Figure 2A** |  |  | **KM curve**  **Log-Rank test**  **Propensity score weighting method** |  |  | NOAC = 3,396  Warfarin = 5,197  No Treatment = 9,898 |
| NOAC | 4.964 | 5,505 |  | 0.952 (vs. W)  <0.001 (vs. No Tx) | 0.419 (vs. W)  <0.001 (vs. No Tx) |  |
| Warfarin | 4.989 | 5.875 |  | <0.001 (vs. No Tx) | < 0.001 (vs. No Tx) |  |
| No treatment | 8.651 | 9.395 |  |  |  |  |
| **Figure 2B** |  |  |  |  |  |  |
| NOAC | 2.800 | 2.258 |  | 0.520 (vs. W)  0.236 (vs. No Tx) | <0.001 (vs. W)  0.904 (vs. No Tx) |  |
| Warfarin | 2.999 | 3.919 |  | <0.001 (vs. No Tx) | <0.001 (vs. No Tx) |  |
| No treatment | 2.463 | 2.226 |  |  |  |  |
| **Figure 2C** |  |  |  |  |  |  |
| NOAC | 7.764 | 5.505 |  | 0.663 (vs. W)  <0.001 (vs. No Tx) | <0.001 (vs. W)  <0.001 (vs. No Tx) |  |
| Warfarin | 7.988 | 5.875 |  | <0.001 (vs. No Tx) | <0.001 (vs. No Tx) |  |
| No treatment | 11.113 | 9.395 |  |  |  |  |

**Figure 3**

| **Figure 3** | **Hazard ratio**  **(vs. No treatment)** | **95% CI** | **Statistical method** | **P value**  **(vs. No treatment)** | **# sample** |
| --- | --- | --- | --- | --- | --- |
| **Efficacy** |  |  | **Cox regression**  **Forest plot** |  |  |
| No treatment | Ref | Ref |  | Ref | 9,898 |
| aTTR < 30% | 0.92 | [0.79-1.07] |  | 0.254 | 1,768 |
| aTTR 30-50% | 0.60 | [0.48-0.74] |  | < 0.001 | 913 |
| aTTR 30-50% | 0.43 | [0.35-0.54] |  | < 0.001 | 1,220 |
| aTTR > 70% | 0.38 | [0.30-0.49] |  | < 0.001 | 1,296 |
| NOAC | 0.39 | [0.33-0.45] |  | < 0.001 | 3,396 |
| **Safety** |  |  |  |  |  |
| No treatment | Ref | Ref |  | Ref | 9,898 |
| aTTR < 30% | 2.32 | [1.89-2.87] |  | < 0.001 | 1,768 |
| aTTR 30-50% | 1.49 | [1.10-1.99] |  | 0.007 | 913 |
| aTTR 30-50% | 1.02 | [0.76-1.37] |  | 0.905 | 1,220 |
| aTTR > 70% | 1.06 | [0.78-1.44] |  | 0.702 | 1,296 |
| NOAC | 0.86 | [0.69-1.08] |  | 0.192 | 3,396 |
| **Composite outcome** |  |  |  |  |  |
| No treatment | Ref | Ref |  | Ref | 9,898 |
| aTTR < 30% | 1.19 | [1.06-1.35] |  | 0.003 | 1,768 |
| aTTR 30-50% | 0.78 | [0.65-0.92] |  | 0.004 | 913 |
| aTTR 30-50% | 0.55 | [0.46-0.66] |  | < 0.001 | 1,220 |
| aTTR > 70% | 0.52 | [0.43-0.63] |  | < 0.001 | 1,296 |
| NOAC | 0.49 | [0.43-0.55] |  | < 0.001 | 3,396 |

**Figure 4**

| **Figure 4** | **Hazard ratio**  **(vs. No treatment)** | **95% CI** | **Statistical method** | **P value**  **(vs. No treatment)** | **# sample** |
| --- | --- | --- | --- | --- | --- |
| **Efficacy** |  |  | **Cox regression**  **Forest plot** |  |  |
| No treatment | Ref | Ref |  | Ref | 9,898 |
| TTR < 30% | 0.61 | [0.53-0.69] |  | < 0.001 | 3,038 |
| TTR 30-50% | 0.67 | [0.55-0.82] |  | < 0.001 | 1,067 |
| TTR 30-50% | 0.50 | [0.38-0.66] |  | < 0.001 | 643 |
| TTR > 70% | 0.52 | [0.38-0.72] |  | < 0.001 | 449 |
| NOAC | 0.38 | [0.33-0.45] |  | < 0.001 | 3,396 |
| **Safety** |  |  |  |  |  |
| No treatment | Ref | Ref |  | Ref | 9,898 |
| TTR < 30% | 1.70 | [1.40-2.05] |  | < 0.001 | 3,038 |
| TTR 30-50% | 1.14 | [0.84-1.55] |  | 0.411 | 1,067 |
| TTR 30-50% | 1.31 | [0.91-1.88] |  | 0.152 | 643 |
| TTR > 70% | 1.47 | [0.98-2.20] |  | 0.064 | 449 |
| NOAC | 0.85 | [0.68-1.06] |  | 0.149 | 3,396 |
| **Composite outcome** |  |  |  |  |  |
| No treatment | Ref | Ref |  | Ref | 9,898 |
| TTR < 30% | 0.82 | [0.74-0.92] |  | < 0.001 | 3,038 |
| TTR 30-50% | 0.77 | [0.65-0.92] |  | 0.003 | 1,067 |
| TTR 30-50% | 0.66 | [0.53-0.82] |  | < 0.001 | 643 |
| TTR > 70% | 0.71 | [0.55-0.91] |  | 0.007 | 449 |
| NOAC | 0.48 | [0.42-0.55] |  | < 0.001 | 3,396 |

**S1 FIGURE**

| **S1 Figure** | **Hazard ratio**  **(vs. No treatment)** | **95% CI** | **Statistical method** | **P value**  **(vs. No treatment)** | **# sample** |
| --- | --- | --- | --- | --- | --- |
| **Ischemic stroke/systemic embolsim** |  |  | **Cox regression**  **Forest plot** |  |  |
| No treatment | Ref | Ref |  | Ref | 9,898 |
| aTTR < 30% | 0.94 | [0.82-1.26] |  | 0.483 | 1,768 |
| aTTR 30-50% | 0.66 | [0.51-0.93] |  | 0.001 | 913 |
| aTTR 30-50% | 0.49 | [0.39-0.73] |  | < 0.001 | 1,220 |
| aTTR > 70% | 0.44 | [0.39-0.70] |  | < 0.001 | 1,296 |
| NOAC | 0.50 | [0.42-0.60] |  | < 0.001 | 3,396 |
| **Critical care bleeding** |  |  |  |  |  |
| No treatment | Ref | Ref |  | Ref | 9,898 |
| aTTR < 30% | 6.79 | [3.79-12.17] |  | < 0.001 | 1,768 |
| aTTR 30-50% | 5.79 | [2.96-11.30] |  | < 0.001 | 913 |
| aTTR 30-50% | 3.59 | [1.79-7.27] |  | < 0.001 | 1,220 |
| aTTR > 70% | 3.00 | [1.41-6.42] |  | 0.004 | 1,296 |
| NOAC | 1.41 | [0.69-3.07] |  | 0.391 | 3,396 |

**S2 FIGURE**

| **S2 Figure** | **Hazard ratio**  **(vs. No treatment)** | **95% CI** | **Statistical method** | **P value**  **(vs. No treatment)** | **# sample** |
| --- | --- | --- | --- | --- | --- |
| **Efficacy** |  |  | **Cox regression**  **Forest plot** |  |  |
| No treatment | Ref | Ref |  | Ref | 9,898 |
| aTTR < 30% | 0.92 | [0.79-1.06] |  | 0.254 | 1,768 |
| aTTR 30-50% | 0.59 | [0.48-0.74] |  | < 0.001 | 913 |
| aTTR 30-50% | 0.43 | [0.35-0.54] |  | < 0.001 | 1,220 |
| aTTR > 70% | 0.38 | [0.30-0.49] |  | < 0.001 | 1,296 |
| Low-dose NOAC | 0.38 | [0.33-0.45] |  | < 0.001 | 2,690 |
| Standard-dose NOAC | 0.42 | [0.31-0.57] |  | < 0.001 | 706 |
| **Safety** |  |  |  |  |  |
| No treatment | Ref | Ref |  | Ref | 9,898 |
| aTTR < 30% | 2.31 | [1.88-2.85] |  | < 0.001 | 1,768 |
| aTTR 30-50% | 1.48 | [1.11-1.98] |  | 0.008 | 913 |
| aTTR 30-50% | 1.01 | [0.75-1.37] |  | 0.927 | 1,220 |
| aTTR > 70% | 1.05 | [0.78-1.42] |  | 0.739 | 1,296 |
| Low-dose NOAC | 0.84 | [0.66-1.07] |  | 0.165 | 2,690 |
| Standard-dose NOAC | 0.95 | [0.62-1.44] |  | 0.793 | 706 |
| **Composite outcome** |  |  |  |  |  |
| No treatment | Ref | Ref |  | Ref | 9,898 |
| aTTR < 30% | 1.20 | [1.06-1.35] |  | 0.004 | 1,768 |
| aTTR 30-50% | 0.78 | [0.65-0.92] |  | 0.004 | 913 |
| aTTR 30-50% | 0.55 | [0.46-0.65] |  | < 0.001 | 1,220 |
| aTTR > 70% | 0.52 | [0.43-0.63] |  | < 0.001 | 1,296 |
| Low-dose NOAC | 0.48 | [0.43-0.55] |  | < 0.001 | 2,690 |
| Standard-dose NOAC | 0.53 | [0.41-0.68] |  | < 0.001 | 706 |

**S3 FIGURE**

| **S3 Figure** | **Hazard ratio**  **(vs. No treatment)** | **95% CI** | **Statistical method** | **P value**  **(vs. No treatment)** | **# sample** |
| --- | --- | --- | --- | --- | --- |
| **Efficacy** |  |  | **Cox regression**  **Forest plot** |  |  |
| No treatment | Ref | Ref |  | Ref | 9,898 |
| aTTR < 30% | 0.92 | [0.79-1.06] |  | 0.244 | 1,768 |
| aTTR 30-50% | 0.59 | [0.48-0.74] |  | < 0.001 | 913 |
| aTTR 30-50% | 0.43 | [0.35-0.54] |  | < 0.001 | 1,220 |
| aTTR > 70% | 0.38 | [0.30-0.49] |  | < 0.001 | 1,296 |
| Apixaban | 0.34 | [0.13-0.91] |  | 0.031 | 184 |
| Dabigatran | 0.39 | [0.32-0.48] |  | < 0.001 | 1,468 |
| Rivaroxaban | 0.38 | [0.31-0.48] |  | < 0.001 | 1,744 |
| **Safety** |  |  |  |  |  |
| No treatment | Ref | Ref |  | Ref | 9,898 |
| aTTR < 30% | 2.31 | [1.88-2.85] |  | < 0.001 | 1,768 |
| aTTR 30-50% | 1.48 | [1.11-1.98] |  | 0.008 | 913 |
| aTTR 30-50% | 1.01 | [0.75-1.37] |  | 0.927 | 1,220 |
| aTTR > 70% | 1.05 | [0.78-1.42] |  | 0.739 | 1,296 |
| Apixaban | 0.88 | [0.66-3.55] |  | 0.856 | 184 |
| Dabigatran | 0.81 | [0.62-1.07] |  | 0.141 | 1,468 |
| Rivaroxaban | 0.93 | [0.69-1.26] |  | 0.638 | 1,744 |
| **Composite outcome** |  |  |  |  |  |
| No treatment | Ref | Ref |  | Ref | 9,898 |
| aTTR < 30% | 1.20 | [1.06-1.35] |  | 0.004 | 1,768 |
| aTTR 30-50% | 0.78 | [0.65-0.92] |  | 0.004 | 913 |
| aTTR 30-50% | 0.55 | [0.46-0.65] |  | < 0.001 | 1,220 |
| aTTR > 70% | 0.52 | [0.43-0.63] |  | < 0.001 | 1,296 |
| Apixaban | 0.44 | [0.20-0.98] |  | 0.045 | 184 |
| Dabigatran | 0.48 | [0.41-0.56] |  | < 0.001 | 1,468 |
| Rivaroxaban | 0.49 | [0.41-0.59] |  | < 0.001 | 1,744 |

**S4 FIGURE**

| **Figure 4** | **Hazard ratio**  **(vs. No treatment)** | **95% CI** | **Statistical method** | **P value**  **(vs. No treatment)** | **# sample** |
| --- | --- | --- | --- | --- | --- |
| **Efficacy** |  |  | **Cox regression**  **Forest plot** |  |  |
| No treatment | Ref | Ref |  | Ref | 4,878 |
| aTTR < 30% | 0.78 | [0.63-0.96] |  | 0.019 | 1,170 |
| aTTR 30-50% | 0.55 | [0.41-0.74] |  | < 0.001 | 519 |
| aTTR 30-50% | 0.42 | [0.31-0.58] |  | < 0.001 | 662 |
| aTTR > 70% | 0.42 | [0.31-0.56] |  | < 0.001 | 844 |
| NOAC | 0.39 | [0.33-0.46] |  | < 0.001 | 3,396 |
| **Safety** |  |  |  |  |  |
| No treatment | Ref | Ref |  | Ref | 4,878 |
| aTTR < 30% | 2.40 | [1.82-3.18] |  | < 0.001 | 1,170 |
| aTTR 30-50% | 1.60 | [1.08-2.37] |  | 0.019 | 519 |
| aTTR 30-50% | 0.91 | [0.58-1.44] |  | 0.690 | 662 |
| aTTR > 70% | 1.23 | [0.85-1.80] |  | 0.277 | 844 |
| NOAC | 0.97 | [0.74-1.26] |  | 0.799 | 3,396 |
| **Composite outcome** |  |  |  |  |  |
| No treatment | Ref | Ref |  | Ref | 4,878 |
| aTTR < 30% | 1.10 | [0.93-1.30] |  | 0.259 | 1,170 |
| aTTR 30-50% | 0.76 | [0.60-0.96] |  | 0.021 | 519 |
| aTTR 30-50% | 0.52 | [0.41-0.68] |  | < 0.001 | 662 |
| aTTR > 70% | 0.58 | [0.46-0.73] |  | < 0.001 | 844 |
| NOAC | 0.50 | [0.43-0.58] |  | < 0.001 | 3,396 |
